# Supplementary material for: Anxiolytic-like Effects of the Positive GABAB Receptor Modulator GS39783 Correlate with Mice’s Individual Basal Anxiety and Stress Reactivity
Source: Pharmaceuticals (Basel). 2022 Feb 16;15(2):233. doi: 10.3390/ph15020233 (PMC8878184; doi:10.3390/ph15020233)
Supplement: Supplementary file 1 [file pharmaceuticals-15-00233-s001.zip › pharmaceuticals-1581975-supplementary.pdf]

SUPPLEMENTARY EXPERIMENTS

Supplementary experiment 1: GS39783 effects in the elevated plus maze

Methods

Subjects and treatments: 15 male and 27 female mice were used. Animals were tested only once, 30 min after treatment with either 0, 10, or 30 mg/kg GS39783.

Setup: A traditional elevated plus maze for mice was used (arm dimensions: 94 cm long and 12 cm wide, 21 cm high walls, elevation: 69 cm). The illumination of the dark arms was 180-210 lux and of the open arms 290-320 lux. A camera installed above the maze recorded the behavior of the mice. After each experiment, the maze was cleaned with water.

Testing procedure: The mice were put into the center of the EPM facing an open arm. The mice could freely move for 5 min and animals' behavior was recorded with the installed camera. Using a tracking software (EthoVision XT, version 11, Noldus Information Technology, Wageningen, The Netherlands), the latency to enter the open arm, the percentage of time spent in the open arms, the percentage of arm entries into the open arm and the total distance travelled were analyzed.

Results

Figure S1 depicts the mean latencies to enter the open arms of the elevated plus maze, the percent time spent in the open arms, the percent entries to the open arms, and the total distance travelled after treatment with 0, 10 and 30 mg/kg GS39783. An ANOVA using sex and treatment as between-subject factors revealed, for all behavioral measures, neither main effects of sex (Fs < 0.50, Ps > 0.50) or of treatment (Fs < 1.40, Ps > 0.24), nor interactions between sex and treatment (Fs < 1.37, Ps > 0.25). This indicates that GS39783 has no general anxiolytic-like effects on behaviors expressed on the elevated plus maze.

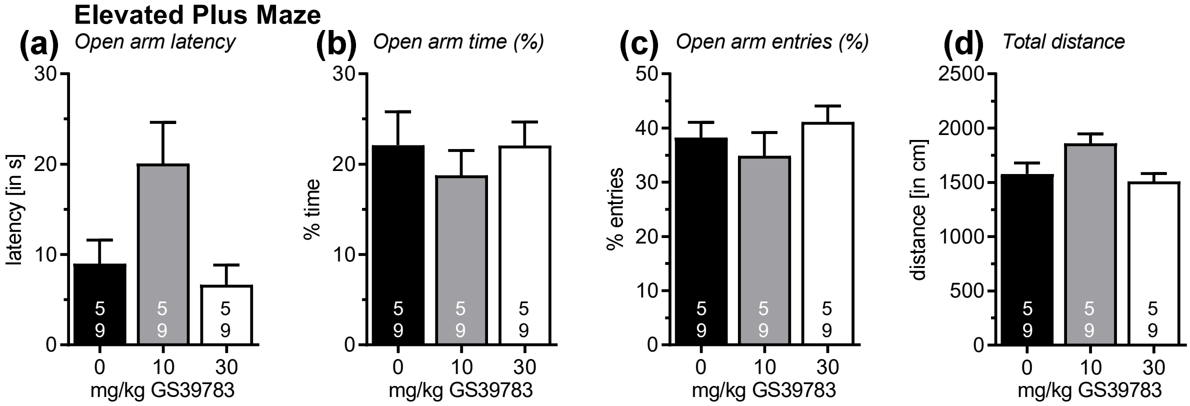

Figure S1. *Bar diagrams showing different behavioral measures of the elevated plus maze test. GS39783 treatment did not affects the latency to enter the open arm (a), the time spent on the open arm (b), the percent entries on the open arm (c) and the total distance travelled (d). Depicted are means + SEM; data were analyzed by ANOVAs. Sexes were pooled since there were no effects of sex (numbers in the bars represent group sizes for females and males).*

Supplementary experiment 2: GS39783 effects in the odor avoidance test

Methods

Subjects and treatments: 16 male mice were subjected to the test. Animals were tested twice on two consecutive days and were treated with 0 or 30 mg/kg GS39783 in a randomized order.

Setup: Two hole-board boxes equipped with an infrared beam/sensor system that was able to detect hole visits were used (ActiMot2 Hole-Board System, TSE Systems, Bad Homburg, Germany). Boxes were constructed from transparent Plexiglas with the dimensions of 51.5 cm × 51.5 cm × 41 cm. The hole-board had 16 holes with a diameter of 1.5 cm each. Holes were categorized into four corner and eight wall holes (each two between two neighboring corners). The four center holes were closed with lids. The setup was in an experimental room with an illumination of 290-320 lux.

Odors: Mice were exposed to urine samples of cows, coyotes and female mice, as well as tap water as a control. Cow urine samples were acquired from the Institute of Genetics and Animal Breeding, Polish Academy of Sciences, Jastrzębiec. Coyote urine was bought from Maine Outdoor Solutions Inc., (Hermon, ME, USA). Female

mice urine was collected in our laboratory by placing female mice in a metabolic cage (Tecniplast, Hohenpeißenberg, Germany). Urine samples from different female mice were mixed, i.e. all estrus phases were presented. Urine samples were stored at in 4° C (cow and coyote) or at -20°C (female mice) until use.

Testing procedure: To present odors, 1 ml urine samples or water were pipetted into glass bowls (4 cm outer diameter, 2.5 cm height) which were placed ca. 1-2 cm underneath the corner holes. In each test, all four different samples were presented but the location of the different odor samples was pseudo-randomized. For the experiment, the mice were put in the center of the setup and were then allowed to freely explore the setup. The hole visits of the mice were automatically detected by infrared detectors (visit duration > 300 ms). After 30 min, the animals were put back into the home cages and the boxes were cleaned with 70% alcohol.

Results

In the hole board, avoidance/approach behavior to corner holes with samples of water (control), urine samples of female mice, cows and coyotes were tested. Treatment with 30 mg/kg GS39783 increased total hole visits (Figure S2a; paired t-test: t_{15} = 2.57, P = 0.02). Due to this increase, the number of visits of the odor holes were normalized to the total number of hole visits (Figure S2b). An ANOVA with odor and treatment as within-subject factors revealed that GS39783 had no main effects ($F_{1,15}$ = 0.52, P = 0.48) but the odors strongly affected the percent hole visits ($F_{3,45}$ = 31.00, P < 0.0001). These two factors did not interact ($F_{3,45}$ = 2.12, P = 0.11). Post-hoc pairwise comparisons showed that in comparison to the hole with a water sample, the holes with samples of cow and coyote urine were less visited (*t*s > 2.50, *P*s < 0.0002). In addition, there were less visits to the holes with samples of coyote urine than those with samples of cow urine (t_{45} = 2.12, P = 0.003). The holes with water and samples of female urine were equally visited (t_{45} = 0.33, P = 0.98).

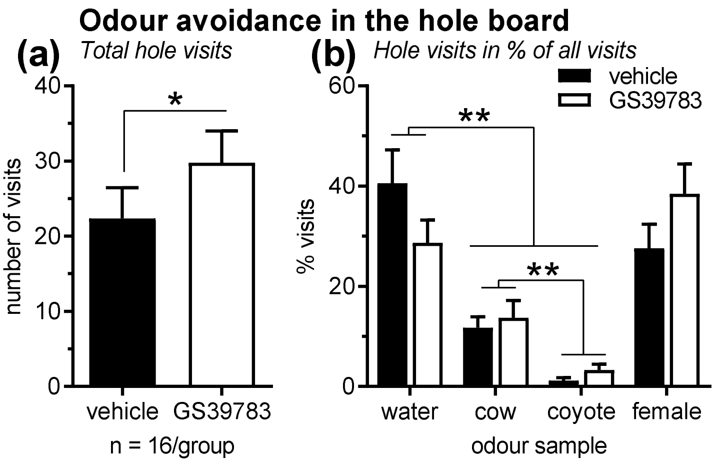

Figure S2. *Bar diagrams showing the effects of GS39783 treatment on hole visits in an odor avoidance task. Total number of hole visits were increased by GS39783 (a). The percent number of visits to holes with samples of cow and coyote urine was generally decreased (b) indicating an avoidance response to these samples. The avoidance to coyote urine samples was stronger than to cow urine samples. These avoidance responses were not affected by GS39783. * P < 0.05, ** P < 0.01, comparisons as indicated.*

Supplementary experiment 3: GS39783 effects on contextual fear and safety conditioning

Methods

Subjects: Twenty-six male mice were used. Each animal was treated either with 0 or 30 mg/kg GS39783 before the retention test.

Setup: Four identical boxes of a computerized-fear conditioning system were used (TSE Systems, Bad Homburg, Germany). Boxes were made of transparent Perspex (46 cm × 46 cm × 32 cm). The boxes had infrared detection frames, loudspeakers for delivering auditory stimuli, house lights and ventilation fans. Illumination of the boxes was 4 lux and the background noise was 55 dB SPL. The floors of the boxes consisted of stainless-steel grids (diameter of 4 mm, distance between bars: 9 mm) which were connected to a shock unit. Delivery of the electric stimuli were controlled by a TSE

software. This software also measured the time the mice spent with freezing. Freezing was defined as no further interception of infrared beams for more than a second. Several studies showed that there is a high correlation between observational scoring of freezing and the software's detection of freezing.

Testing procedure: On the first day of the experiment, animals were habituated to the transparent boxes for a duration of 2.5 min. On the next consecutive two days, two contextual fear and safety conditioning sessions were performed. In each session, the animals were placed into the boxes and then exposed to five explicit unpairing of a tone stimulus (10 kHz, 85 dB SPL, 30 s) and a scrambled electric stimulus (0.4 mA, 2 s). Explicit unpairing means that the electric stimuli were applied at pseudo-randomized time points between two tone presentation (mean interstimulus interval: 2 min, range between 1.5 and 2.5 min), with the restriction that the two stimuli were not closer than 30 s. On the fourth day, the animals were treated and 30 min later, a retention test on conditioned contextual fear and safety was conducted. Hence, animals were put into the boxes and 30 s later, five tone stimuli were presented to animals at interstimulus intervals of one minute.

Results

In this experiment, freezing was measured to assess the mice's freezing response to the conditioning context (which was associated with the administration of electric stimuli) and to the safety CS (which was associated with the absence of the electric stimuli). Treatment with 30 mg/kg GS39783 neither affected freezing to the conditioning context nor the decrease of freezing during presentations of the safety CS (Figure S3a; treatment: $F_{1,24} = 0.17$, $P = 0.68$; safety CS: $F_{1,24} = 20.56$, $P < 0.0001$; interaction: $F_{1,24} = 0.41$, $P = 0.53$). This was supported by the analysis of the percent change (inhibition) of freezing behavior by the safety CS (Figure S3b; $t_{24} = 0.66$, $P = 0.52$).

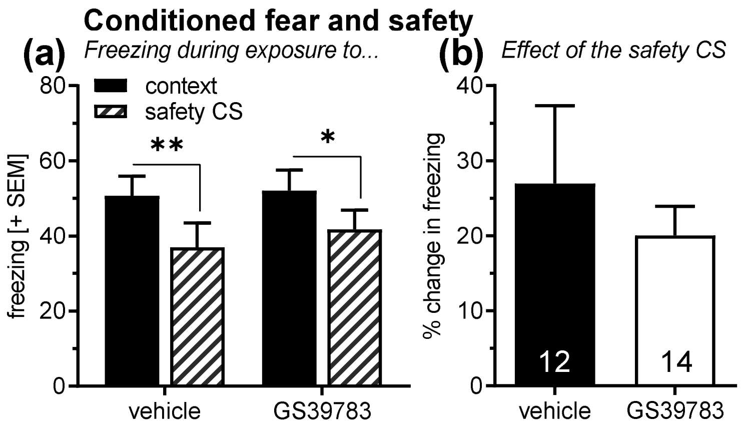

Figure S3. *Bar diagrams depicting the percent freezing behavior to the conditioning context and during presentations of the safety CS (a), as well as the percent change (inhibition) of freezing by the safety CS (b). The safety CS reduced freezing behavior. However, 30 mg/kg GS39783 did neither affect freezing behavior to the context nor the effect of the safety CS. ** P < 0.01, * P < 0.05, comparisons as indicated.*

Supplementary experiment 4: GS39783 effects on sociability test

Methods

Subjects and treatments: 36 male animals were used. Animals were injected with 0 or 30 mg/kg GS39783 and 30 min later, they were tested for sociability.

Setup: The test was adapted from Moy et al. (2004). Two boxes with dimensions of 23 cm × 48 cm × 33 cm, made of dark grey PVC were used. In the opposite corners of each box, two small cylindrical wire cages were located (diameter: 8.5 cm, height: 10 cm). Bottles were put on the top of the wired cages to prevent the test mice climbing on them. Behavior of the test mice were recorded by a camera installed above the box.

Testing Procedure: The test consisted of two 10-min phases. In the first phase, habituation, the test mouse was put in the center of the box and could freely explore the box and empty wire cages. In the second phase, sociability test, a stranger mouse that has had no previous contact to the test mouse was put in one of the wire cages (localization of the stranger was pseudo-randomized). Then, the test mouse could

again freely explore the box. Videos were analyzed by a video tracking software (EthoVision XT, version 11, Noldus Information Technology, Wageningen, The Netherlands) using nose point detection of animals. The duration of nose entries into an area of 3 cm around both wire cages was measured. Before each test, the boxes and wire cages were cleaned with 70% alcohol.

Results

In the sociability test, the nose contacts of the test mice with the small wire mesh cages were measured (Figure S4a). An ANOVA with phase (habituation, sociability) and cage (with and without stranger) as within-subject factors and treatment as between-subject factors revealed that in the sociability phase the cage with the stranger was more approached than the empty cage (interaction phase x cage: $F_{1,34}$ = 20.74, P < 0.0001). Treatment with 30 mg/kg GS39783 did not interfere with this sociability (interaction treatment x phase x cage: $F_{1,34}$ = 0.62, P = 0.44). Notably, GS39783 treatment had a tendency to generally reduce contacts with the cages ($F_{1,34}$ = 3.39, P = 0.07). Analysis of the sociability indices confirmed these observations (Figure S4b). The indices increased during the sociability phase ($F_{1,34}$ = 28.91, P < 0.0001) and this increase was not affected by GS39783 treatment ($F_{1,34}$ = 1.09, P = 0.30).

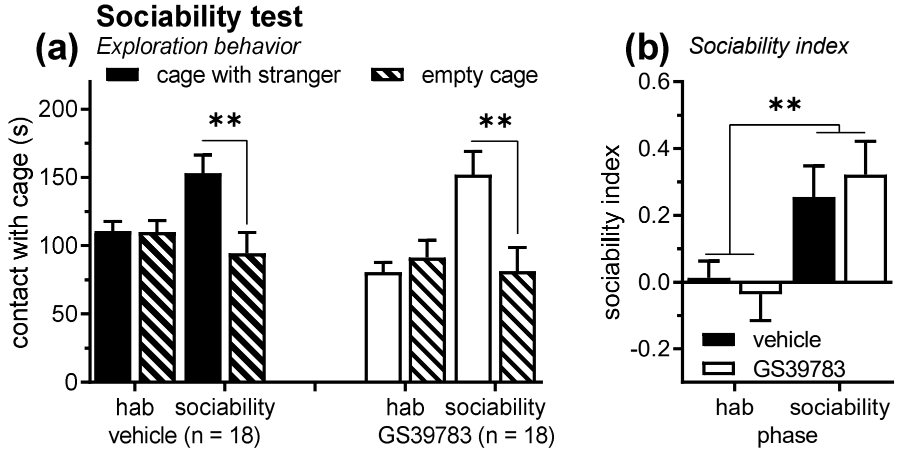

Figure S4. *Bar diagrams showing the effects of 30 mg/kg GS39783 treatment on exploration behavior, i.e. cage contacts (a), and sociability indices (b). In the habituation phase, none of the empty cages were preferred. In the sociability phase, the mice had more contacts with the cage including the stranger than with the empty cage showing sociability. This sociability was not affected by GS39783 treatment. ** P < 0.01, comparisons as indicated.*
